# Effect of light duration and variation on the growth and reproductive performance in breeder geese

**DOI:** 10.5713/ab.25.0349

**Published:** 2025-08-25

**Authors:** Min Jung Lin, Shen Chang Chang, Li Jen Lin, Jhih Siang Chang, Shao Yu Peng, Tzu Tai Lee

**Affiliations:** 1 Bachelor of Program in Scientific Agriculture, National Pingtung University of Science and Technology, Pingtung, Taiwan; 2 Department of Animal Science, National Chiayi University, Chiayi, Taiwan; 3 Southern Region Branch, Taiwan Livestock Research Institute, Ministry of Agriculture, Pingtung, Taiwan; 4 School of Chinese Medicine, College of Chinese Medicine, China Medical University, Taichung, Taiwan; 5 Department of Animal Science, National Chung Hsing University, Taichung, Taiwan; 6 Department of Animal Science, National Pingtung University of Science and Technology, Pingtung, Taiwan; 7 The iEGG and Animal Biotechnology Center, National Chung Hsing University, Taichung, Taiwan; 8 Smart Sustainable New Agriculture Research Center (SMARTer), Taichung, Taiwan

**Keywords:** Breeder Geese, Growth Performance, Reproductive Performance, Time and Change of Light

## Abstract

**Objective:**

This study aims to investigate the effects of time and light variation on the growth and reproductive performances of breeder geese.

**Methods:**

A total of 32 ganders and 96 dames in their first laying season, with an average age of 10 months, were assigned to eight rooms, each containing four ganders and twelve dams. A split-plot design was used, incorporating two prelay photoperiod treatments: a 7-hour light period (P7H), and a gradually decreasing light (GDL) group. Two variable photoperiod schedules were applied as sub-treatments and randomized across the rooms. In one treatment, the egg production rate in breeder geese decreased to an average below 30%, after which the photoperiod increased by 15 minutes each week (change time). A fixed photoperiod of nine hours was maintained (fixed time) until the end of the egg-laying period.

**Results:**

Geese in the GDL light group had a longer laying duration than P7H light group (243.75 vs 191.75 days; p<0.01). Number of eggs per goose in the GDL light group showed a trend toward higher values than P7H light group (81.82 vs 55.45 egg/bird; p = 0.0779). However, the fertility and hatchability in GDL light group were significantly lower than the P7H light group at all periods, respectively (48.35 vs. 62.57% and 42.80 vs. 53.17%; p<0.05). Income over feed cost for the GDL and P7H light groups was 3,069.6 and 2,535.5 NT$/bird, respectively.

**Conclusion:**

Geese exposed to a 12-hour light (12L:12D) regimen during the pre-laying period exhibited a longer laying duration and higher egg production per bird. However, maintaining a fixed lighting schedule of 9 hours of light and 15 hours of darkness (9L:15D) after the peak laying period is recommended to optimize production profitability by supporting better fertility and hatchability.

## INTRODUCTION

Geese can be classified into three egg-laying types based on latitude [1–3]. Geese exhibit seasonal reproduction in a natural light environment [4–6]. In Taiwan, egg-laying begins in November and ends in May of the following year [7,8]. The photoperiod stimulates the onset of egg production and helps sustain it [9,10]. This seasonal reproductive pattern is primarily controlled by the pineal gland and hypothalamic-pituitary-gonadal (HPG) axis, which respond to variations in daylight duration. The secretion of hormones such as melatonin, prolactin, luteinizing hormone (LH), and follicle-stimulating hormone (FSH) fluctuates according to light exposure, impacting fertility and reproductive behavior [11–16]. The reproduction of breeding geese is affected by the light source, spectrum, and intensity, and regimen of light. Light regulation technology is widely used to stimulate egg production in breeding geese by manipulating these factors [17–23].

Photoperiod influences changes in the negative feedback control of steroid hormones on gonadotropin, affecting the age at first egg in poultry [24,25]. Geese exposed to a 19-hour light regimen exhibited suppressed egg production and induced molting in breeding geese [21]. During the pre-laying period, light regulation stimulates egg production before the onset of laying in breeding geese. When the photoperiod exceeds 14 hours, egg production in breeder geese decreases [26]. At 8-month-old White Roman geese under 12-hour, 8-hour, and 16-hour light treatments began laying eggs after about one month, four months, and exhibited suppressed egg production, respectively [27]. Geese exposed to a 14-hour photoperiod for 21 weeks experienced suppressed laying activity. Supplementary lighting between 12.0 to 13.5 hours during pre-laying resulted in geese in the supplementary lighting group laying 47.6 eggs per goose, compared to 26.4 eggs per goose in the natural lighting group [25]. Pre-laying light regimen of 6.5 hours induced breeding geese to start laying eggs earlier. Although this treatment increased the number of eggs produced before the peak laying period, overall egg production per goose remained unchanged throughout the entire laying period [28]. Landes geese in China were exposed to an 18-hour light period. After 2 to 2.5 months, the photoperiod was reduced to 11 hours, which enabled the geese to start laying eggs [29].

The effective photoperiod for stimulating an earlier initiation of egg production falls within a narrow range between 8 and 14 hours of light. Photoperiod not only plays a crucial role in initiating egg production but also in maintaining it. During the laying period, geese exposed to 8-hour and 10-hour light treatments exhibited longer laying periods, higher egg weights, and increased laying rates compared to those under a 12-hour light regimen, with the 10-hour treatment resulting in the highest laying rate [30]. Furthermore, on average, geese under a 9-hour light treatment laid 18 more eggs per goose than those exposed to 11-hour light [31,32]. Geese under a 9-hour light treatment produced 29 more eggs than those under a 13-hour light regimen. Although a longer photoperiod, such as a 13-hour light treatment during the egg production period, can initiate an earlier start to laying and a more intensive laying rate, it also results in a shorter laying period (71 to 80 days less), ultimately reducing total egg production [33].

Geese’s seasonal reproduction involves hormonal regulation that influences the induction of egg production.Geese exposed to 12-hour light treatments had higher melatonin levels at night (5,250 pg/pineal gland) compared to daytime (2,257 pg/pineal gland) [34]. In Magang ganders, plasma testosterone levels ranged from 0–1 ng/mL during the non-breeding season (April to June) and increased to 5–10 ng/mL during the breeding season. Long photoperiods stimulate the secretion of prolactin, which inhibits LH secretion [35]. In female geese, plasma estradiol (E2) levels were positively correlated with triglyceride levels during the egg-laying period, indicating that both increased with egg production [36]. Geese under 20-hour light treatments showed a decline in progesterone (P4) and estradiol (E2) levels with increasing age, which corresponded to a decrease in egg production [37]. Therefore, this study aims to optimize reproductive induction and maintain performance by manipulating light duration and variation in breeder geese.

## MATERIALS AND METHODS

### Animals and experiment design

**Figure 1:**
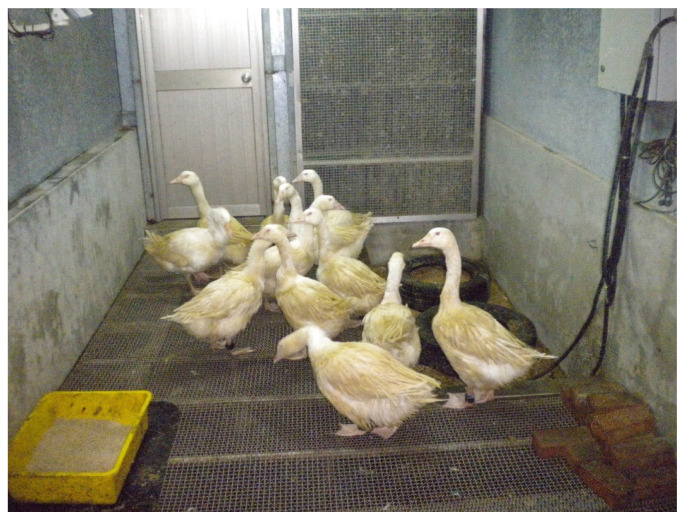
Geese were subjected to different photoperiod treatments during the pre-laying period, followed by either fixed or variable light schedules during the laying period.

The care and use of all geese followed the Regulations of Laboratory Animals at the Changhua Animal Propagation Station, Livestock Research Institute, Council of Agriculture, Taiwan. The study was approved by the Institutional Animal Care and Use Committee (IACUC) at Changhua, Taiwan (LRICHIACUP 10212). Geese were reared under white light-emitting diode lighting. Light intensity was maintained at 30 lux at eye level of standing geese (Figure 1).

**Figure 2:**
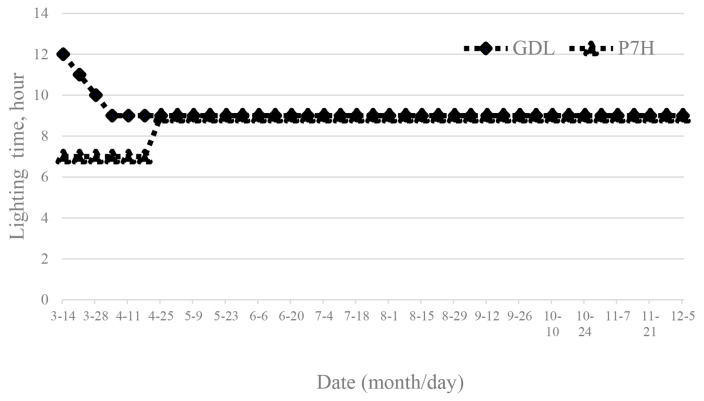
Different photoperiods during prelaying stage in White Roman Goose. GDL: the light duration was initially set at 12 hours and then reduced by 1 hour per week; P7H group, the photoperiod was maintained at 7 hours for the first six weeks light. Both treatment groups were maintained under a 9-hour photoperiod per day.

A total of 32 ganders and 96 dames in their first laying season, with an average age of 10 months, were allotted eight rooms. Each room housed four ganders and twelve dams. The daily sunshine duration in Changhua ranged from 11 hours to 58 minutes on March 14. A split-plot design included two prelaying photoperiods, i.e. 7-hour light (7L:17D) period (P7H) and a 12-hour light (12L:12D) period (P12H), regarded as the main treatments randomized over four rooms in an environmentally controlled house (ECH), and two variable photoperiod schedules, i.e. photoperiod was increased by 15 minutes each week (CHP), while the other maintained a fixed photoperiod of nine hours (FIXP), regarded as sub-treatments and randomized over four pens in each of the four rooms. The four replications for the prelaying photoperiod treatments and for variable photoperiod schedules treatments. Both treatment groups were maintained under a 9-hour photoperiod per day. These treatments were randomized across eight rooms in an ECH. Two variable photoperiod schedules, i.e. the egg production rate in breeder geese fell to an average below 30% (Figure 2).

Two variable photoperiod schedules were applied when the egg production rate in breeder geese dropped below an average of 30%. In one schedule, the photoperiod was increased by 15 minutes each week (change time, CHP), while the other maintained a fixed photoperiod of nine hours (FIXP) until the end of the laying period. These schedules were regarded as sub-treatments and were randomized across four pens in each of the four rooms at the Northern Region Branch (23°51’N, 120°33’E), MOA-TLRI, Taiwan. The prelay photoperiod treatments were replicated twice for the variable photoperiod schedules treatments.

Once the geese were moved into an ECH, they were provided with a restricted diet containing 13.0% crude protein (CP) and 2,350 kcal/kg metabolizable energy (ME). Each bird received 150 g of resting rations (Table 1). The photoperiod was adjusted to 9 hours of light and 15 hours of darkness (9L:15D). The laying geese were fed *ad libitum* with a laying diet containing 18.0% CP and 2,700 kcal/kg ME. Each room measured 2.4 m×6.5 m (15.6 m^2^), with a stocking density of 0.975 birds per m^2^. Upon entry into the ECH, breeder geese were subjected to restrict feeding with 150 g of resting rations per bird per day. When the photoperiod was adjusted to 9 hours of light and 15 hours of darkness (9L:15D), the feed was switched to a layer diet and provided *ad libitum*. This feeding regimen was designed to prevent rapid development of the female reproductive system during the photostimulation phase, which could otherwise lead to premature egg production. At this point, the male reproductive system may not be fully matured, potentially resulting in reduced fertilization rates during the early laying period.

### Egg production performances

All goslings were wing-banded immediately after hatching and raised in a temperature-controlled house. From 12 weeks of age, the geese were subjected to restricted feeding, with each bird receiving 150 g of a resting ration per day (containing 13.0% CP and 2,350 kcal/kg ME). At an average age of 10 months, the birds were allocated to eight rooms. The average body weight of the geese at allocation ranged from 2.2 to 4.61 kg per bird and was evenly distributed across pens. Statistical analysis was conducted immediately after allocation to confirm no significant differences in body weight among treatment groups, ensuring homogeneity at the start of the experiment. Body weight and feed consumption were measured at the peak and end of the laying period, immediately after the birds were transferred into the ECH. The peak of egg production (PK) was defined as the average laying rate over two consecutive days, reaching 30% for all the geese. Egg production ceased (EPC) at approximately 35 weeks after the geese entered the house. The EPC was defined as the average laying rate over two consecutive days dropping to 5% for all the geese.

### Fertilization rate and hatchability

A total of 9,244 eggs were collected from 20 batches. To assess the fertilization rate, hatchability, and gosling output, eggs were gathered in batches every two weeks. Incubation was conducted using an automatic incubator (Tonz Nan Incubators Manufactured), with temperature settings of 37.7°C from days 0 to 14, 37.5°C from days 15 to 28, and 37.2°C during hatching. Fertilized eggs were identified by visual inspection on day 7 of incubation. The fertilization rate was calculated as the percentage of fertilized eggs among the total incubated eggs. Hatchability was defined as the percentage of goslings hatched from the total number of incubated eggs, whereas the hatchability of fertilized eggs was determined as the percentage of goslings hatched from fertilized eggs.

### Serum biochemical parameters

During the experiment periods, four geese (two male and two female) per room were randomly selected for serum sample collection on the first day of each month. Blood samples were processed approximately 4 to 5 hours after collection by centrifugation at 3,000×g for 10 minutes at 4°C. Serum samples were stored at −4°C for up to one day before analysis. Serum biochemical parameters were analyzed using an automatic biochemical analyzer (7150 auto-analyzer; Hitachi). For reproductive hormones assay, the serum was analyzed using electrochemiluminescence immunoassay with an Elecsys and Cobase immunoassay analyzer (UniCel DxI 800 Access Immunoassay System; Beckman Coulter).

### Reproductive gland analysis

At the end of the study, one female and one male goose from each room were slaughtered, and their carcass traits—including pre-slaughter body weight, reproductive tract length, ovary weight, and follicle diameters—were measured. The ovaries were removed at the time of slaughter, and their size and numbers were measured. The length and weight of the reproductive tract were measured from the fimbria to the cloacal opening. On the other hand, ovarian weight refers to the weight of ovarian follicles. The male geese were slaughtered, and their carcass traits, including pre-slaughter and testicular weight, were recorded.

### Statistical analysis

The data collected were statistically analyzed using the MIXED procedure of SAS software [38] following a split-plot design. Two prelaying photoperiod treatments were randomized across four rooms regarded as main plots, while two laying alternative light photoperiod treatments were randomized across four pens regarded as split plots in each room. Data on the prelaying photoperiod treatments and dietary protein contents were subjected to analysis of variance using the Statistical Analysis System Institute Package (SAS). The mean values were compared using the LSMEANS procedure, with significance set at p<0.05.

The mathematic model was:


(1) $${\text{Y}}_{\text{ijkl}}=\mu +{\text{T}}_{\text{i}}+\gamma_{\text{ij}}+{\text{P}}_{\text{k}}+{\left(\text{T}\times \text{P}\right)}_{\text{ik}}+{ɛ}_{\text{ijkl}}$$

where Y_ijkl_ is the measurement of the average of birds in pen *l*, room *j*, subjected to laying alternative light photoperiod *k* and prelaying photoperiod treatment *i*; μ is the overall mean; T_i_ is the fixed effect of prelaying photoperiod treatment *i*; γ_ij_ is the residual term, where γ_ij_ Ç N (0, σ^2^_γ_); P_k_ is the fixed effect of laying alternative light photoperiod treatment *k*; (T×P)_ik_ is the two-way interaction between prelaying photoperiod treatment *i* and laying alternative light photoperiod *k*; ɛ_ijkl_ is the residual term, where ɛ_ijkl_ Ç N (0, σ^2^_ɛ_).

## RESULTS

### Growth performance

The effects of light duration before egg production and changes in light duration during the egg-laying period on the growth performance of breeder geese are presented in Table 2. The geese subjected to a gradually decreasing light treatment (GDL) during the prelaying period exhibited significantly higher body weight than those under a 7-hour lighting treatment (P7H) at the peak of egg production in the prelaying period (4.72 vs. 4.96 kg/bird; p<0.05). There were no significant differences in body weight, body weight gain, and feed consumption between geese exposed to a photoperiod that changed by 15 minutes each week (change time, CHP) and those maintained under a fixed 9-hour photoperiod (FIXP) during the egg-laying period.

### Reproductive performance

The effects of light duration before egg production and changes in light duration during the egg-laying period on the reproductive characteristics of breeder geese are presented in Table 3. The geese in the GDL light group exhibited a significantly longer laying duration than those in the P7H light group (243.75 vs. 191.75 days; p<0.01). The egg number per goose in the GDL light group tended to be higher than in the P7H light group (81.82 vs 55.45 egg/bird; p<0.1). However, the fertility and hatchability were significantly lower in the GDL light group than those of the P7H light group across all periods (48.35 vs. 62.57%; p<0.05 and 42.80 vs. 53.17%; p<0.05, respectively). During the initial stage of egg production (0% to 30%; S1), the geese in the GDL light group exhibited a significantly higher geese-day egg production rate than those in the P7H light group at the stage of egg production from 0 to 30% (S1) (17.25 vs 4.00%; p<0.01). However, at the subsequent stage (egg production >30% to <30%; S2), the fertility and hatchability remained significantly lower in the GDL light group than those in the P7H light group (49.58 vs. 74.59%; p<0.001 and 43.82 vs. 65.04%; p<0.001, respectively).

Similarly, the fertility and hatchability in the CHP light group were significantly lower than those in the FIXP light group across all periods (44.55 vs. 66.37%; p<0.01 and 37.85 vs. 58.11%; p<0.01, respectively). At the S2 stage, the fertility and hatchability in the CHP light group were significantly lower than those in the FIXP light group (54.74 vs. 69.43%; p<0.001 and 47.12 vs. 61.74%; p<0.001, respectively). Furthermore, at the final stage of egg production (30% down to 5%; S3), the fertility and hatchability in the CHP light group were significantly lower than those in the FIXP light group (26.11 vs. 62.60%; p<0.001 and 21.56 vs. 53.30%; p<0.001, respectively).

### Serum biochemical parameters

The mean corpuscular hemoglobin (MCH) level (Haemoglobin/Erythrocyte) in the GDL light group was significantly higher than those in the P7H light group (61.12 vs. 58.88 pg; p<0.05, Table 4). MCH, which refers to the average amount of hemoglobin in a single red blood cell, is essential for maintaining optimal egg production in breeder hens [39]. Similarly, the MCH levels in the CHP light group were significantly lower than those in the FIXP light group (58.99 vs. 61.01 pg; p<0.05). Additionally, the low-density lipoprotein (LDL) level in the CHP light group was significantly lower than those in the FIXP light group (43.00 vs. 58.94 pg; p<0.05). Hens with higher blood LDL concentrations may experience a reduction in total egg production, whereas highly productive hens tend to exhibit lower LDL levels [40]. These findings highlight the complexity of lipid metabolism and its association with egg production in poultry.

### Reproductive gland analysis

During the onset of the reproductive phase, both ganders and geese exhibited significant increases in testicular size and genital tract length, as well as ovarian weight [41]. Photoperiod manipulation in breeder geese can regulate the hypothalamus and pituitary gland, thereby influencing the stimulation and refractoriness of the reproductive system. Enhanced laying performance, particularly in out-of-season conditions, has been associated with elevated expression levels of FSHβ and LHβ subunit mRNA [42]. No significant difference in carcass characteristics was observed between P7H and GDL light groups (Table 5). Similarly, there were no significant differences in carcass characteristics between the CHP and FIXP light groups.

### Relation of the reproduction characteristics and serum biochemical parameters

**Figure 3:**
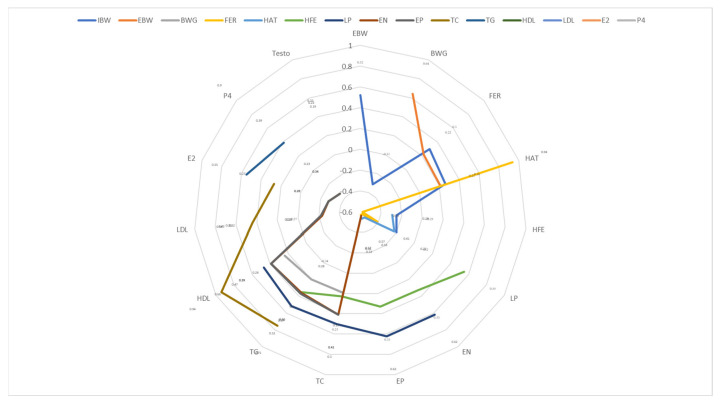
Partial correlation coefficients growth performance, and reproduction characteristics in breeder geese. IBW, initial of body weight (kg/bird); EBW, ending of body weight (kg/bird); BWG, body weight gain (kg/bird); FER, fertility (%); HAT, hatchability (%); HFE, hatchability of fertilized eggs (%); LP, laying period (days); EN, egg number per goose (egg/geese); EP, egg production (%); TC, total cholesterol (mg/dL); TG, triglycerides (mg/dL); HDL, high density lipoprotein (mg/dL); LDL, low density lipoprotein (mg/dL); E2, estradiol (pg/mL); P4, progesterone (ng/mL); Testo, testosterone (ng/mL).

The laying period in this study was positively correlated with egg number per goose (EN), egg production (EP, %), total cholesterol level (TC, mg/dL), and triglycerides level (TG, mg/dL) and high-density lipoprotein level (HDL, mg/dL) was 0.62 (p<0.001), 0.62 (p<0.001), 0.50 (p<0.001), 0.52 (p*<*0.001) and 0.47 (p*<*0.001), respectively (Figure 3). EN was negatively correlated with estradiol level (E2, pg/mL) and progesterone level (P4, ng/mL), with correlation coefficients of −0.28 (p*<*0.01) and −0.34 (p*<*0.001), respectively. Additionally, testosterone levels (ng/mL) were positively correlated with fertility rate and hatchability rates, with correlation coefficients of 0.23 (p*<*0.05) and 0.25 (p*<*0.01), respectively.

### Economic benefit

The economic benefits of light duration before egg production and changes in light duration during the egg-laying period are summarized in Table 6. Evaluation of the outcomes and economic performance of time and change of light in breeder geese (Table 7). The geese in the GDL light group had a longer laying duration than those in the P7H light group. The GDL light group in egg number per goose tended to be higher than in the P7H light group. However, the fertility and hatchability were significantly lower in the GDL light group than those of the P7H light group across all periods. The feed cost for the GDL and P7H light groups during the experimental period was 782.5 and 707.6 NT$/bird, respectively. The income over feed cost (IOFC) for the GDL and P7H light groups were 3,069.6 and 2,535.5 NT$/bird, respectively. Similarly, the IOFC for the CHP and FIXP lights group was 2,298.7 and 3,340.3 NT$/bird, respectively.

## DISCUSSION

The poultry sector is an important livestock sector that involves numerous risks in the wide production and management chain, starting from farm establishment and extending to disease prevention, animal nutrition and animal husbandry [43–45]. The goose sector is a branch of livestock farming that has an important place worldwide and is carried out for both meat and feather production. Shi et al [1] indicated that the breeding season of breeder geese varies by latitude, with distinct reproductive performances observed in high (40º to 45º N), medium (30º to 40º N), and low (22º to 25º N) latitudes. These differences are primarily influenced by photoperiod, which affects goose behavior and reproductive performance via pituitary secretion of gonadotropins and prolactin [16].

Taiwan, located between 22° and 25° N and 120° and 122° E, is classified as a low-latitude region (22º to 25º N). In Taipei (approximately 25.037° N), the daily sunshine duration ranges from 10 hours 35 minutes (June) to 13 hours 42 minutes (December), while in Pingtung (approximately 22.54° N), the sunshine duration ranges from 10 hours 45 minutes (June) to 13 hours 32 minutes (December) [46]. The egg-laying period for breeder geese in Taiwan begins in November and ends in May of the following year [7].

A 7L:17D lighting regime for six weeks to induce egg-laying, followed by a shift to 9L:15D during the laying period, delayed egg-laying onset by approximately one month [23]. This lighting adjustment technique successfully induced breeder geese to lay 48.5–51.1 eggs per parity [47]. In Guangdong Province, China, a low-latitude region, breeder geese exhibit a low-latitude reproductive pattern [1]. Maintaining a photoperiod of 18L:4D for 2 to 2.5 months during winter (December to January), followed by a transition to 11L:13D, successfully induced out-of-season laying [29].

Seasonal birds are primarily influenced by photoperiod, which plays a crucial role in regulating reproductive activity. When birds enter the photosensitive phase, the HPG axis is activated through the neuroendocrine system. Gonadotropin-releasing hormone stimulates the release of FSH and LH, which in turn promote gametogenesis and reproductive behaviors [48]. In this study, the use of the GDL light and P7H light groups successfully induced egg-laying in breeder geese (Table 3). However, breeder geese under the GDL light group had a laying duration of 243.75 days, which was 52 days longer than those under the P7H light group (Table 3).

Geese exposed to a 19-hour light prelaying light period had a longer laying period than those exposed to a 6.5-hour prelaying light period [20]. However, the fertility and hatchability performance was reversed, with geese under the 19-hour light prelay period exhibiting lower fertility and hatchability. This study observed similar results, as geese in the GDL light group exhibited significantly lower fertility and hatchability than those in the P7H light group throughout the entire period (Table 3). Additionally, geese in the GDL light group consumed more feed than those in the P7H light group (Table 3). This increased feed consumption may be attributed to the prolonged egg-laying period, which could lead to a decline in both gander semen quality and breeder egg quality, ultimately reducing fertility and hatchability.

During the laying period, geese under a 9-hour lighting schedule laid 18 more eggs than those under an 11-hour lighting schedule and 29 more eggs than those under a 13-hour lighting schedule. Although a 13-hour lighting schedule can stimulate earlier egg-laying, the laying period is 71–80 days shorter than that under a 9-hour lighting schedule [33]. This finding is consistent with previous research indicating that longer photoperiods are associated with extended laying periods but tend to result in lower fertility and hatchability compared to shorter photoperiods [13]. A similar pattern was observed in the fertilization rate of hatching eggs gradually increased at the onset of laying, remained high during peak production, and then gradually declined as laying progressed [49]. In this study, the increased feed consumption may be attributed to the prolonged egg-laying period, which could negatively affect the breeder egg quality, ultimately leading to reduced fertility and hatchability. This study suggests that hens should receive sufficient light supplementation from the beginning of egg production until 50 weeks to balance their photorefractoriness [50]. The method of increasing light duration during the laying period has been referenced in subsequent studies [51–53]. Broiler breeders, increasing the photoperiod in 15-minute or 1-hour increments from 11 to 16 hours during the laying cycle could mitigate the decline in the laying rate [51]. At 33 weeks, broiler breeders were subjected to an increase in photoperiod to 18L:6D, while the other half remained at 14L:10D. The results showed that increasing the photoperiod had no significant effect on reproductive performance and ovarian morphology, likely because the breeders had already reached peak egg production and sexual maturity [52]. Therefore, the decline in fertilization rate after the peak laying period may be due to the ineffectiveness of increasing lighting post-peak. In this study, although no difference was observed in the laying rate between the CHP and the FIXP light groups, the fertility and hatchability were significantly lower in the CHP light group than those in the FIXP light group throughout all periods (Table 3). Additionally, in the CHP group, fertility and hatchability declined significantly from the stage of egg production of >30% to <30% (S2) and further from 30% down to 5% (S3). Goose egg yolk contains a higher level of LDL compared to chicken egg yolk [53]. Elevated LDL levels have been associated with maintaining the integrity of the spermatozoa plasma membrane [54]. In this study, blood LDL levels were positively correlated with both egg fertility and hatchability, as was testosterone concentration (Table 6). These findings suggest that higher circulating LDL levels may contribute to improved sperm membrane integrity, thereby enhancing fertilization and hatching success. Geese in the CHP light group had lower LDL levels and testosterone concentrations than the FIXP light group (Table 4). Although testicle weight, ovary weight, and follicle length in the CHP group were not significantly different from those in the FIXP light group, their average values were lower (Table 5). When the egg production rate in breeder geese fell below 30%, a photoperiod increase of 15 minutes per week was implemented. However, since the geese had already reached peak egg production, this adjustment did not enhance egg production. The reduced fertility and hatchability in the CHP light group may have been influenced by changes in blood composition and hormone levels.

Production costs can be categorized into direct and indirect expenses. Direct costs include gosling expenses, feed costs, labor costs, medical expenses, energy costs, and material costs. In contrast, indirect costs consist of depreciation expenses for goose housing and equipment. Since rearing conditions vary across farms, direct comparisons of production costs are challenging. Feed costs were calculated based on raw material prices at the time, with the cost per kilogram of resting and laying diets being 10.41 and 12.83 NTD$, respectively (Tables 6, 7). Geese subjected to GDL lighting treatment during the pre-laying period had a higher IOFC. However, geese in the FIXP light group exhibited better IOFC during the laying period. Therefore, applying GDL lighting treatment before egg production and maintaining the FIXP lighting schedule during the laying period is recommended to maximize profitability.

## CONCLUSION

This study demonstrated that geese exposed to a 12-hour light (12L:12D) regimen during the pre-laying period exhibited a longer laying duration and higher egg production per bird. However, maintaining a fixed lighting schedule of 9 hours of light and 15 hours of darkness (9L:15D) after the peak laying period is recommended to optimize production profitability by supporting better fertility and hatchability.

## Figures and Tables

**Table 1:** Ingredients and nutrient compositions of the experimental diets (as fed basis)

Ingredients	Resting diet	Laying diet
Yellow corn	50.35	57.35
Soybean meal	12.7	25.7
Wheat bran	20.0	-
Rice bran	10.0	-
Alfalfa meal	-	2.0
Fish meal	-	2.5
Oyster shell	-	3.5
Molasses	3.0	3.0
Salt	0.3	0.3
Dicalcium phosphate	1.9	3.5
Limestone, pulverized	1.3	1.7
Choline chloride, 50%	0.1	0.1
DL-Methionine	0.1	0.15
Vitamin premix^1)^	0.1	0.1
Mineral premix^2)^	0.15	0.1
Total	100	100
Calculated values		
Crude protein (%)	13.0	18.0
ME (kcal/kg)	2,350	2,700
Total calcium (%)	1.02	3.11
Available phosphorus (%)	0.35	0.50

1) Resting diet was supplied per kilogram of diet: vitamin A, 10,000 IU; vitamin D3, 2,000 IU; vitamin E, 20 mg; vitamin K3, 1.5 mg; vitamin B1, 1.00 mg; vitamin B2, 4.8 mg; vitamin B6, 3.00 mg; vitamin B12, 16 μg; folic acid, 0.50 mg; calcium pantothenate, 10.0 mg; niacin, 25 mg; Biotin, 2.00 mg; Fe (FeSO_4_), 120 mg; Cu (CuSO_4_.5H_2_O), 22.5 mg; Mn (MnSO_4_·H_2_O), 120 mg; Zn (ZnO), 75.0 mg; I (KI), 1.275 mg; Co (CoSO_4_), 0.375 mg; Se (Na_2_SeO_3_), 0.27 mg.

2) Laying diet was supplied per kilogram of diet: vitamin A, 10,000 IU; vitamin D3, 2,000 IU; vitamin E, 20 mg; vitamin K3, 1.5 mg; vitamin B1, 1.00 mg; vitamin B2, 4.8 mg; vitamin B6, 3.00 mg; vitamin B12, 16 μg; folic acid, 0.50 mg; calcium pantothenate, 10.0 mg; niacin, 25 mg; Biotin, 2.00 mg; Fe (FeSO_4_), 80.0 mg; Cu (CuSO_4_·5H_2_O), 15.0 mg; Mn (MnSO_4_·H_2_O), 80.0 mg; Zn (ZnO), 50.0 mg; I (KI), 0.85 mg; Co (CoSO_4_), 0.25 mg; Se (Na_2_SeO_3_), 0.18 mg.

**Table 2:** Effect of time and change of light on the growth performances in breeder geese

Item	Time of light			Change of light		
	
	GDL	P7H	SEM	CHP	FIXP	SEM
Body weight (kg/bird)						
EH (kg)^1)^	3.34^a^	3.23^a^	0.061	3.31	3.26	0.061
PK (kg)	4.96^a^	4.72^b^	0.057	4.84	4.84	0.057
EPC (kg)	5.28^a^	5.06^a^	0.192	5.06	5.27	0.192
Body weight gain (kg/bird)						
From EH to PK	1.66^a^	1.49^a^	0.066	1.56	1.59	0.066
From PK to EPC	0.28^a^	0.34^a^	0.146	0.22	0.40	0.146
From EH to EPC	1.97^a^	1.83^a^	0.176	1.78	2.01	0.176
Feed consumption (kg feed/bird)	60.97^a^	56.27^a^	3.336	57.12	60.12	3.336
Feed efficiency (kg feed/egg)	0.76^a^	1.05^a^	0.099	0.84	0.98	0.099

Results are given as the means of 4 rooms.

GDL: the light duration was initially set at 12 hours and then reduced by 1 hour per week; P7H group, the photoperiod was maintained at 7 hours for the first six weeks light; CHP: the egg production rate in breeder geese fell to an average below 30%, increased the photoperiod by 15 minutes each week; FIXP: maintained a fixed photoperiod of 9 hours until the end of the egg-laying period.

1) EH: the time point when the birds entered the house. PK: approximately 15 week from enter geese house, representing an average of 2 d in regard to the laying rate, reached 30% among the entire rooms. EPC: approximately 38 week from enter geese house, it represented a drop of 5% compared to an average laying rate of 2 d among the entire rooms.

a,b Means without the same superscripts within the same row under treatment differ significantly (p<0.05).

SEM, standard error of means for treatment.

**Table 3:** Effect of time and change of light on reproductive characteristics in breeder geese

Item	Time of light			Change of light		
	
	GDL	P7H	SEM	CHP	FIXP	SEM
All period						
Laying duration (d)	243.75^a^	191.75^b^	5.031	216.25^x^	219.25^x^	5.031
Egg number (egg/bird)	81.82^a^	55.45^a^	7.991	73.83^x^	63.44^x^	7.991
Geese-day egg production (%)	32.55^a^	22.13^a^	3.189	29.38^x^	25.30^x^	3.189
Fertility (%)	48.35^b^	62.57^a^	2.314	44.55^y^	66.37^x^	2.314
Hatchability (%)	42.80^b^	53.17^a^	2.253	37.85^y^	58.11^x^	2.253
Hatchability of fertilized eggs (%)	85.86^a^	85.70^a^	2.022	84.04^x^	87.53^x^	2.022
S1: stage of egg production from 0% to 30%						
Laying duration (d)	34.50^a^	17.00^a^	9.881	17.00^x^	34.50^x^	9.881
Egg number (egg/bird)	5.58^a^	3.27^a^	0.853	3.42^x^	5.44^x^	0.853
Geese-day egg production (%)	17.25^a^	4.00^b^	1.425	10.00^x^	11.25^x^	1.425
Fertility (%)	59.04^a^	57.57^a^	9.007	50.83^x^	65.78^x^	9.007
Hatchability (%)	53.19^a^	51.17^a^	9.586	43.65^x^	60.71^x^	9.586
Hatchability of fertilized eggs (%)	85.94^a^	87.67^a^	5.699	81.82^x^	91.79^x^	5.699
S2: stage of egg production from>30% to <30%						
Laying duration (d)	122.25^a^	84.50^a^	13.366	125.50^x^	81.25^x^	13.366
Egg number (egg/bird)	53.27^a^	34.10^a^	9.147	53.81^x^	33.56^x^	9.147
Geese-day egg production (%)	39.25^a^	39.50^a^	2.942	41.50^x^	37.25^x^	2.942
Fertility (%)	49.58^b^	74.59^a^	1.066	54.74^y^	69.43^x^	1.066
Hatchability (%)	43.82^b^	65.04^a^	1.093	47.12^y^	61.74^x^	1.093
Hatchability of fertilized eggs (%)	86.67^a^	87.18^a^	1.017	84.81^x^	89.03^x^	1.017
S3: stage of egg production from 30% to 5%						
Laying duration (d)	87.00^a^	90.25^a^	17.37	73.75^x^	103.50^x^	17.37
Egg number (egg/bird)	23.00^a^	18.07^a^	2.692	16.60^x^	24.47^x^	2.692
Geese-day egg production (%)	26.25^a^	21.75^a^	2.883	24.50^x^	23.50^x^	2.883
Fertility (%)	37.53^a^	51.18^a^	5.041	26.11^y^	62.60^x^	5.041
Hatchability (%)	32.88^a^	41.98^a^	4.008	21.56^y^	53.30^x^	4.008
Hatchability of fertilized eggs (%)	85.34^a^	84.76^a^	1.707	84.78^x^	85.32^x^	1.707

Results are given as the means of 4 rooms.

GDL: the light duration was initially set at 12 hours and then reduced by 1 hour per week; P7H group, the photoperiod was maintained at 7 hours for the first six weeks light; CHP: the egg production rate in breeder geese fell to an average below 30%, increased the photoperiod by 15 minutes each week; FIXP: maintained a fixed photoperiod of 9 hours until the end of the egg-laying period.

a,b Means without the same superscripts within the same row under time of light treatment differ significantly (p<0.05).

x,y Means without the same superscripts within the same row under change of light treatment differ significantly (p<0.05).

SEM, standard error of means for treatment.

**Table 4:** Effect of time and change of light on the blood biochemical parameters in breeder geese

Item	Time of light			Change of light		
	
	GDL	P7H	SEM	CHP	FIXP	SEM
WBC (10^3^/μL)	278.81^a^	280.39^a^	2.755	280.76^x^	278.44^x^	2.755
RBC (10^6^/μL)	1.84^a^	1.94^a^	0.058	1.91^x^	1.87^x^	0.058
HB (g/dL)	11.13^a^	11.41^a^	0.361	11.23^x^	11.32^x^	0.361
HT (%)	33.56^a^	34.87^a^	0.950	34.33^x^	34.09^x^	0.950
MCV (fL)	182.93^a^	180.13^a^	0.788	180.26^x^	182.79^x^	0.788
MCH (pg)	61.12^a^	58.88^b^	0.463	58.99^y^	61.01^x^	0.463
MCHC (g/dL)	33.37^a^	32.69^a^	0.363	32.71^x^	33.36^x^	0.363
PLT (10^3^/μL)	7.25^a^	9.94^a^	2.052	11.81^x^	5.38^x^	2.052
GLU (mg/dL)	176.19^a^	172.31^a^	10.418	189.13^x^	159.38^x^	10.418
BUN (mg/dL)	2.00^a^	1.94^a^	0.099	2.06^x^	1.88^x^	0.099
CREA (mg/dL)	0.25^a^	0.26^a^	0.005	0.26^x^	0.25^x^	0.005
UA (mg/dL)	3.09^a^	3.63^a^	0.482	3.65^x^	3.06^x^	0.482
GOT (U/L)	25.00^a^	30.69^a^	4.073	25.69^x^	30.00^x^	4.073
GPT (U/L)	9.69^a^	10.00^a^	0.645	9.13^x^	10.56^x^	0.645
TP (g/dL)	5.11^a^	4.89^a^	0.341	4.88^x^	5.13^x^	0.341
ALB (g/dL)	2.13^a^	1.95^a^	0.116	1.94^x^	2.13^x^	0.116
GLO (g/dL)	2.99^a^	2.94^a^	0.243	2.94^x^	2.99^x^	0.243
A/G	0.72^a^	0.68^a^	0.034	0.68^x^	0.72^x^	0.034
TC (mg/dL)	165.94^a^	164.38^a^	13.244	157.75^x^	172.56^x^	13.244
TG (mg/dL)	699.00^a^	225.00^a^	255.277	518.69^x^	405.31^x^	255.277
HDL (mg/dL)	89.94^a^	84.38^a^	10.094	86.06^x^	88.25^x^	10.094
LDL (mg/dL)	47.00^a^	54.94^a^	2.490	43.00^y^	58.94^x^	2.490
Estradiol (pg/mL)	89.05^a^	58.23^a^	20.060	65.20 ^x^	82.08 ^x^	20.060
Progesterone (ng/mL)	0.56^a^	0.20^a^	0.296	0.17^x^	0.60^x^	0.296
Testosterone (ng/mL)	0.38^a^	0.31^a^	0.205	0.18^x^	0.51^x^	0.205

Results are given as the means of 4 rooms.

GDL: the light duration was initially set at 12 hours and then reduced by 1 hour per week; P7H group, the photoperiod was maintained at 7 hours for the first six weeks light; CHP: the egg production rate in breeder geese fell to an average below 30%, increased the photoperiod by 15 minutes each week; FIXP: maintained a fixed photoperiod of 9 hours until the end of the egg-laying period.

a,b Means without the same superscripts within the same row under time of light treatment differ significantly (p<0.05).

x,y Means without the same superscripts within the same row under change of light treatment differ significantly (p<0.05).

SEM, standard error of means for treatment; WBC, white blood cell; RBC, erythrocyte; HB, haemoglobin; HT, hematocrit; MCV, HT/RBC; MCH, HB/RBC; MCHC, HB/HT; PLT, platelet; GLU, glucose; BUN, blood urea nitrogen; CREA, creatinine; UA, uric acid; GOT, glutamyl oxaloacetic transaminase; GPT, glutamyl pyrubic transaminase; TP, total protein; ALB, albumin; GLO, globulin; A/G, albumin/globulin; TG, triglycerides; TC, total cholesterol; HDL, high density lipoprotein; LDL, low density lipoprotein.

**Table 5:** Effect of time and change of light on the carcass characteristics in breeder geese

Item	Time of light			Change of light		
	
	GDL	P7H	SEM	CHP	FIXP	SEM
Carcass weight (kg/bird)	5.60^a^	5.10^a^	0.299	5.51^x^	5.19^x^	0.299
Abdominal fat pad weight	225.56^a^	163.75^a^	45.543	235.75^x^	153.56^x^	45.543
Liver weight (g/bird)	129.88^a^	109.63^a^	11.098	122.06^x^	117.44^x^	11.098
Gizzard weight (g/bird)	153.50^a^	161.40^a^	9.896	167.56^x^	147.3^x^	9.896
Heart weight (g/bird)	36.75^a^	32.44^a^	1.580	35.75^x^	33.44^x^	1.580
Intestinal weight (g/bird)	301.50^a^	253.50^a^	34.951	307.50^x^	247.50^x^	34.951
Length of genital tract (cm/bird)	51.00^a^	52.75^a^	13.371	30.25^x^	73.50^x^	16.376
Testicle weight (g/bird)	7.24^a^	6.52^a^	2.322	4.75^x^	9.01^x^	2.322
Ovary weight (g/bird)	50.88^a^	17.00^a^	27.280	5.50^x^	62.38^x^	27.280
Length of follicle (cm)	12.93^a^	7.25^a^	7.263	0.00^x^	20.18^x^	7.263

Results are given as the means of 4 rooms.

GDL: the light duration was initially set at 12 hours and then reduced by 1 hour per week; P7H group, the photoperiod was maintained at 7 hours for the first six weeks light; CHP: the egg production rate in breeder geese fell to an average below 30%, increased the photoperiod by 15 minutes each week; FIXP: maintained a fixed photoperiod of 9 hours until the end of the egg-laying period.

a Means without the same superscripts within the same row under time of light treatment differ significantly (p<0.05).

x Means without the same superscripts within the same row under change of light treatment differ significantly (p<0.05).

SEM, standard error of means for treatment.

**Table 6:** Evaluation of the economic benefit of time and change of light in breeder geese

Item	Time of light		Change of light	
	
	GDL	P7H	CHP	FIXP
Feed consumption in resting diet (kg/bird)	0	6.016	3.008	3.008
Feed consumption in laying diet (kg/bird)	60.97	50.25	57.96	53.26
Feed cost (NT$/bird)	782.5	707.6	775.2	714.9
Egg number (egg/bird)	81.82	55.45	73.83	63.44
Hatchability (%)	42.8	53.17	37.85	58.11
Gosling per goose (bird)	35.02	29.48	27.94	36.86
Gosling price (NT$/bird)	110	110	110	110
Gosling income (NT$/bird)	3,852.1	3,243.1	3,073.9	4,055.1
Income over feed cost (NT$/bird)	3,069.6	2,535.5	2,298.7	3,340.3

GDL: the light duration was initially set at 12 hours and then reduced by 1 hour per week; P7H group, the photoperiod was maintained at 7 hours for the first six weeks light; CHP: the egg production rate in breeder geese fell to an average below 30%, increased the photoperiod by 15 minutes each week; FIXP: maintained a fixed photoperiod of 9 hours until the end of the egg-laying period.

Feed cost: based on the costs (NT$/kg) of the ingredients as follows: yellow corn 9.40, soybean meal 17.62, wheat bran 7.38, rice bran 8.00, alfalfa meal 18.98, fish meal 33.00, oyster shell 5.80, cane molasses, 7.85, salt (NaCl) 6.40, dicalcium phosphate 18.50, Limestone, pulverized 2.50, choline chloride, 50% 50.00, DL-Methionine 98.00, vitamin premix 350.00, and mineral premix 15.83. The fees for processing of basal ration per kg were 10.41 for grain mixture of resting diet group. The fees for processing of basal ration per kg were 12.83 for grain mixture of laying diet group.

**Table 7:** Evaluation of the outcomes and economic performance of time and change of light in breeder geese

Item	Time of light		Change of light	
	
	GDL	P7H	CHP	FIXP
Laying duration (d)	243.75^a^	191.75^b^	216.25^y^	219.25^x^
Egg number (egg/bird)	81.82^a^	55.45^b^	73.83^x^	63.44 ^y^
Fertility (%)	48.35^b^	62.57^a^	44.55^y^	66.37^x^
Hatchability (%)	42.80^b^	53.17^a^	37.85^y^	58.11^x^
Feed cost (NT$/bird)	782.5	707.6	775.2	714.9
Income over feed cost (NT$/bird)	3,069.6	2,535.5	2,298.7	3,340.3

GDL: the light duration was initially set at 12 hours and then reduced by 1 hour per week; P7H group, the photoperiod was maintained at 7 hours for the first six weeks light; CHP: the egg production rate in breeder geese fell to an average below 30%, increased the photoperiod by 15 minutes each week; FIXP: maintained a fixed photoperiod of 9 hours until the end of the egg-laying period.

a,b Means without the same superscripts within the same row under time of light treatment differ significantly (p<0.05).

x,y Means without the same superscripts within the same row under change of light treatment differ significantly (p<0.05).
